# Mobile Healthcare for Automatic Driving Sleep-Onset Detection Using Wavelet-Based EEG and Respiration Signals

**DOI:** 10.3390/s141017915

**Published:** 2014-09-26

**Authors:** Boon-Giin Lee, Boon-Leng Lee, Wan-Young Chung

**Affiliations:** 1 Department of Electronic Engineering, Keimyung University, Daegu 704-701, Korea; E-Mail: bglee@kmu.ac.kr; 2 Department of Electronic Engineering, Pukyong National University, Busan 608-737, Korea; E-Mail: boringdragonlee@hotmail.com

**Keywords:** sleep onset, mobile healthcare, electroencephalogram, respiration, adaptive threshold filter, mutual information, wavelet packet transform, support vector machine

## Abstract

Driving drowsiness is a major cause of traffic accidents worldwide and has drawn the attention of researchers in recent decades. This paper presents an application for in-vehicle non-intrusive mobile-device-based automatic detection of driver sleep-onset in real time. The proposed application classifies the driving mental fatigue condition by analyzing the electroencephalogram (EEG) and respiration signals of a driver in the time and frequency domains. Our concept is heavily reliant on mobile technology, particularly remote physiological monitoring using Bluetooth. Respiratory events are gathered, and eight-channel EEG readings are captured from the frontal, central, and parietal (Fpz-Cz, Pz-Oz) regions. EEGs are preprocessed with a Butterworth bandpass filter, and features are subsequently extracted from the filtered EEG signals by employing the wavelet-packet-transform (WPT) method to categorize the signals into four frequency bands: α, β, θ, and δ. A mutual information (MI) technique selects the most descriptive features for further classification. The reduction in the number of prominent features improves the sleep-onset classification speed in the support vector machine (SVM) and results in a high sleep-onset recognition rate. Test results reveal that the combined use of the EEG and respiration signals results in 98.6% recognition accuracy. Our proposed application explores the possibility of processing long-term multi-channel signals.

## 1. Introduction

The rising number of traffic accidents in recent years has become a major issue of concern to society, as well as governments. In particular, road accidents account for 20%–30% of traffic accidents worldwide [1,2]. Fatigue or drowsiness, an intermediate state between wakefulness and sleep that has been defined as a state of impaired alertness associated with a desire or inclination to sleep [3], is a major factor that affects drivers, resulting in slow reaction times, reduced vigilance, and deficits in information processing that cause an irregular driving aptitude. Developing and establishing a system that can accurately detect driving fatigue symptoms can prevent disastrous traffic events. Therefore, extensive studies on methods to prevent traffic accidents have been carried out by automobile manufacturers, as well as research institutes [4,5]. In fact, automobile manufacturers, such as Daimler [6] and Toyota [7], have employed such accident prevention systems. Fatigue detection can be carried out by three methodologies: (1) physiological state monitoring; (2) driving behavior and performance monitoring; or (3) a combination of both.

Given the tremendous increase in the number of mobile device users, mobile-device-based mental fatigue recognition applications could be a new trend in the future. According to [8], Korea has been leading countries worldwide in both overall mobile ownership (99%) and smartphone ownership (67%). Of this 67% of smartphone users, 81% of users are most likely to use their smartphone for applications, and the percentage of users is expected to further increase in the coming years. Our mobile-device-based application aims to encourage more drivers to utilize such systems for their own safety.

For physiological state monitoring, Wu *et al.* [9] combined EEG power spectrum estimation, principal component analysis (PCA), and the fuzzy neural network model to estimate drivers' drowsiness level in a driving simulator. This EEG electrode cap uses signals from 34 channels and ensures satisfactory fatigue measurement accuracy, but it does not perform well in real-time mobile applications. Jung *et al.* [10] employed a similar technique but with artificial neural networks for alertness classification. Meanwhile, Estrada *et al.* [11] implemented a single algorithm to compute the relative EEG spectral powers corresponding to the intersection point between the lower and alpha frequencies at which the performance of the algorithm was unsatisfactory. Murata *et al.* [12] demonstrated that the EEG-α/β, heart rate variability (RRV3), and tracking error tended to increase with the level of drowsiness by calculating the probability using a Bayesian statistical inference algorithm. As an alternative to the EEG-based fatigue estimation methods, other researchers have adopted the use of other physiological signals to monitor the level of driver drowsiness. Hemi *et al.* [13] employed two distinct methods based on eye movement monitoring (with an infrared sensor) and bio-signal processing (with a respiration and HR sensor) to ensure driver safety. However, these studies did not demonstrate the effectiveness of sensors for accurately capturing driver drowsiness related to biomedical signals. Krishnamoorthy *et al.* [14] developed a drowsiness monitoring system to detect the HR and used the variation in HR to predict drowsiness using a photoplethysmograph (PPG) sensor. Wei *et al.* [15] proposed an information-fusion-based drowsiness detection method based on the driver's eye activity, head inclination, sagging posture, heart beat rate, skin electric potential, EEG activities, gripping force on the steering wheel, and lane-keeping characteristics. Among the numerous physiological indicators available to estimate the driver's vigilance level, the EEG signal has been proved to be one of the most predictive and reliable [16] indicator compared to others.

A power spectral analysis of a biomedical signal is one of the most commonly-used approaches to analyze an individual's alertness level. Tanaka *et al.* [17] extracted the frequency-domain features from the R-R interval or HR variability and observed that the time-dependent changes in low frequency (LF), high frequency (HF), and LF/HF correspond to the driver's alertness level. Li *et al.* [18] suggested an HRV frequency analysis method, based on fast Fourier transform (FFT) features, where the HRVs are extracted from the PPG signals. Similar research conducted by Kheder *et al.* [19] proposed an extraction of the HRV analysis features from the wavelet package coefficient with an adaptive threshold method. Takahashi *et al.* [20] estimated the sleepiness level by investigating the physiological response using a multiple linear regression model developed from the indices of ECG (electrocardiogram) and respiration. Concurrently, Hu *et al.* [21] removed the EEG artifact noises by exploiting an independent component analysis with a reference (ICA-R) before extracting the power spectrum features from a 75-channel EEG. Among the EEG-spectrum-related features, 40 features were chosen by SVM-recursive feature elimination to improve the classification accuracy. On the other hand, Zhao *et al.* [22] demonstrated that the wavelet packet energy (WPE) of the EEG signals is strongly correlated with the fatigue level by jointly applying a kernel PCA (KPCA) and an SVM to derive the mental fatigue state.

As is well known, there are currently no reliable and impressive mobile-device-based driver fatigue indicators proposed for monitoring the long-term driver vigilance level. Most of the applications are executed in desktop PCs (and lack mobility) or embedded hardware systems [10,11,13–20]. The restriction of the processing speed and constraints on memory size in embedded hardware practically reduce the fatigue estimation accuracy. Therefore, we propose a mobile-device-based application that takes advantage of both of the approaches of fatigue detection systems while minimizing the constraints. We also propose a wavelet packet transform (WPT) to extract each EEG frequency band from the wavelet coefficient. In this study, the analysis of the respiration signals (regularity) is carried out along with an EEG (time and frequency domains) analysis to achieve a high correlation to the driver's vigilance level. The proposed application consists of three processing phases: preprocessing (Gaussian noise removal), features selection, and classification. Selection of the most descriptive features by a mutual information (MI) approach and investigation of the optimized number of features to increase the classification rate are the most salient objectives of this research.

## 2. System Design

### 2.1. System Architecture

**Figure 1.**
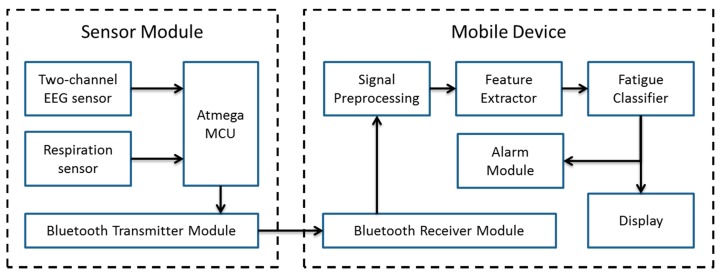
Block diagram of the proposed system architecture for mobile-device-based fatigue indicator application. The system consists of two major parts: (**a**) a sensor module built using an Atmega microprocessor and a Bluetooth module connected to an eight-channel EEG sensor and a respiration sensor and (**b**) a mobile device, in which most of the signal processing takes place. The signals are preprocessed, and features are extracted from clean signals that serve as the input to a classifier. The computed vigilance index is displayed on the mobile screen, and an alarm is triggered if the vigilance index reaches a predefined threshold value. Raw biomedical signals are transmitted via Bluetooth.

Figure 1 illustrates the conceptual design of the proposed mobile-device-based driver fatigue indicator application. It consists of two fundamental parts: a sensing part (sensor module) and an analysis part (mobile device). The sensing part senses raw biomedical signals from a eight-channel EEG sensor (recorded as two referential derivation EEG activity), as well as a respiration sensor. The captured signals are transmitted from the sensors to the mobile device via Bluetooth. The mobile device filters the received raw signals with a Butterworth bandpass filter ranges from 0.1 Hz to 40 Hz. Once the signals are filtered, the most descriptive features can be extracted to serve as input features to a classifier to indicate the driver fatigue level. The features are selected by adopting an MI approach. The resulting fatigue pattern is displayed to the driver. If the fatigue level is critical, an alarm will be triggered by the mobile device, instantly warning the driver. The warning can be provided in various ways, such as an incoming fake call, vibrations, messages, or through external warning devices depending on the driver's preference. The key benefits of our proposed architecture is that no extra display devices or processing units are required to carry out the analysis.

### 2.2. EEG and Respiration Signals

A electroencephalogram (EEG) is brainwave activity that is generally categorized into four typical frequency bands, which are delta (0–4 Hz), theta (4–8 Hz), alpha (8–13 Hz), and beta (13–20 Hz). In the past decades, many studies have been conducted to study the correlation of EEGs with the sleeping pattern [2]. Increased delta activity is observed during the sleep state. On the other hand, the theta wave is a useful indicator to demonstrate an early stage of drowsiness. In addition, for a driver who is sleepy enough to fall asleep, alpha activity increases slowly, but it decreases with concentration or when the driver is in a relaxed wakefulness state. Beta activity decreases during the drowsiness state but increases with a high awareness level of the driver. Therefore, alpha activity is proven to be the most sensitive measure to predict the presence of fatigue, followed by theta activity and delta activity.

Respiration is defined as the transport of oxygen from the outside air to the cells within tissues, and the transport of carbon dioxide occurs in the opposite direction. According to [23], respiratory events may occur throughout non-rapid eye movement (NREM) and rapid eye movement (REM) sleep. This study shows that disordered breathing episodes during NREM sleep are associated with an increased risk of daytime sleepiness. In this study, we aim to determine the effect of respiratory events during driving activities in daytime and night time to monitor the level of drowsiness. Indeed, the foremost objective of this research is to integrate the features extracted from EEGs and respiratory events to accurately indicate the driver fatigue level. Table 1 lists the specifications of the sensor module and mobile device.

The driver fatigue indicator application is developed using a smartphone device that possesses the capabilities of high-speed data transmission (e.g., 3G, 4G LTE), Bluetooth, and Wi-Fi wireless communication and a wide display screen. In this study, Google Nexus 5 is chosen after a detailed review of several smartphones because of its low cost and high-speed operation.

## 3. Materials and Methods

### 3.1. Principle of Wavelet Transform

**Figure 2.**
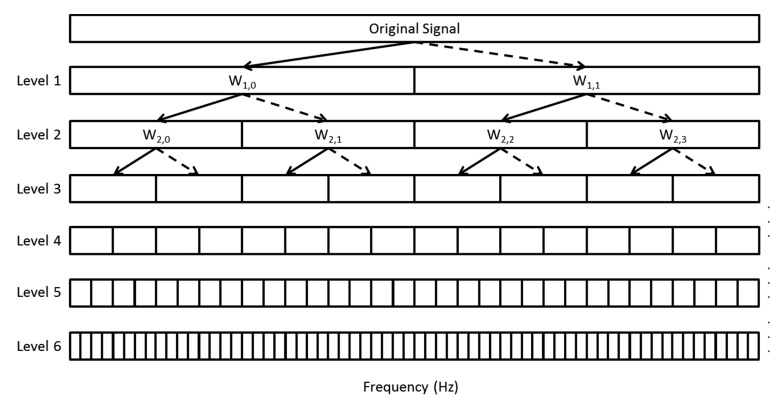
Wavelet packet transform (WPT) decomposed from one to six levels. The original signal is transformed into each frequency component *W_j_*,*_n_* by detail coefficients (solid lines) and scaling coefficients (dotted lines). The frequency indexes range from 0 to 2*^j^* − 1 for zero to the Nyquist frequency (50 Hz (Equation (4)) for WPT in this study) with an original sampling frequency of 100 Hz.

EEGs can be decomposed into four frequency bands with wavelet-packet-transform (WPT) [18] where the time-frequency representation of the EEGs can be obtained, providing better insight in the frequency distribution of EEGs over time. Based on the experiment results on [17], WPT is able to analyze the instantaneous changes in different frequency bands. In overview, WPT demonstrated higher resolution of time-dependent changes in analyzing the signal frequencies than that of DWT and CWT. The wavelet packet decomposition at the *j*-th level of EEG signals gives 2*^j^* sets of sub-band coefficients of length $\left\{P_{j,m}(n)|k=1,2,\ldots ,\frac{N}{2^{j}}\right\}$. These wavelet coefficient vectors reflect the change in the signal with time in the frequency range of:

(1) $$\left[\frac{(m-1)F_{S}}{2^{j+1}},\frac{mF_{S}}{2^{j+1}}\right]$$

where *F_S_* is the sampling frequency, which is 100 Hz in this study and *m* = 0,1, …,2*^j^*^−1^. Figure 2 illustrates the analysis of the original signals with a WPT of levels 1, 2, 3, 4, 5, and 6 used in this study. Meanwhile, other wavelet functions, such as Daubechies D1 (db1) and D4 (db4) [18] are taken into consideration in this study, but the analysis showed that Haar wavelet transform has lower complexity in extracting the frequency band as compared to db1 and db4 to be developed in limited processing capability mobile device. Thus, Haar-based WPT is applied in this study.

On the basis of the frequency bands extracted from the wavelet coefficients, the frequency bands alpha (α), beta (β), theta (θ), and delta (δ) are defined by acquiring the mean of the corresponding frequency bands in level 6, as summarized in Table 2. Each wavelet coefficient in level 6 corresponds to a range of 0.78125 Hz.

As the wavelet *φ_a,b_*(*t*) has an orthogonal basis at *L*^2^(*R*), the *j*-th level power spectrum energy [2] of the wavelet coefficients for each frequency band is calculated by:

(2) $$PWR_{k}=\sum_{k}{\left|d_{k}^{\left(1+1\right)}(n)\right|}^{2}$$

Subsequently, the total power spectrum energy of the frequency bands can be computed by:

(3) $$PWR_{\text{total}}=\sum_{k}PWR_{k}$$

The relative power spectrum energy for the respective frequency bands for resolution level 6, denoted by *RELPWR_k_*, is calculated by:

(4) $$\text{RELPW}R_{k}=\frac{PWR_{k}}{PWR_{\text{total}}}$$

which quantifies the probability distribution of the spectral energy [29]. On the basis of the wavelet packet decomposition described above, multi-dimensional features can be extracted from the two referential derivations EEG activity.

### 3.2. Definition of Mutual Information

MI is a basic concept in information theory that is a good indicator of the relevance between variables and has been used as a measure in several feature selection algorithms [30]. The feature selection algorithm greatly depends on the accuracy of MI [31]. Specifically, in this study, given an input feature *X* extracted from EEGs or respiration signals and the output class *Y* (sleepiness level), the MI between *X* and *Y* can be defined as follows:

(5) I(X;Y)=H(X)+H(Y)−H(X,Y)=H(Y)−H(Y|X)

Where *H*(*X*) and *H*(*Y*) are the marginal entropy of the input feature and the sleepiness level, respectively, and they measure the associated uncertainty. *H*(*X*,*Y*) and *H*(*Y*|*X*) are the joint and conditional entropies of *X* and *Y*. *I*(*X*;*Y*) measures how much the uncertainty of *X* is reduced if *Y* has been observed. If *X* and *Y* are independent, their MI value is zero, e.g., *Y* does not reduce the uncertainty of *X*. Because the sleepiness level is a discrete variable, the entropy *H*(*X*) is defined as:

(6) $$H(X)=-\sum p(X)\log_{2}p(X)$$

Where *p*(*X*) represents the marginal probability distribution of the feature *X*. Maximizing the MI between different features and the desired target can achieve the lowest probability of error [32,33].

### 3.3. Support Vector Machine Classifier

A support vector machine (SVM), developed by Vapnik, is known to be a powerful tool for generating pattern recognition systems with high generalization ability [2]. The basic concept of an SVM involves the adoption of a nonlinear kernel function to transform input features into a high-dimensional feature space and the construction of optimal separating hyperplanes that maximize the margin between the two nearest data points belonging to two separate classes [34]. Nevertheless, the selection of a kernel is critical to ensure that it is associated with the inner product of some nonlinear mapping [35]. To date, typical kernel choices used in BCI research were the Gaussian, polynomial, or radial basis functions.

(7) $$K(x,x_{i})=\exp\left(\frac{{\left\|x-x_{i}\right\|}^{2}}{2\sigma^{2}}\right)$$

For SVMs with RBF kernels, the value of the width or kernel parameter *σ* can be optimally selected by using *n*-fold cross-validation or an independent test set. Many experiments [36–39] have suggested that the number of support vectors (SVs) will vastly increase if the predefined *σ* is too small. Likewise, when *σ* is too large or small, the generalization performance of SVMs will immensely decrease [39]. The fraction of SVs provided an upper bound on the leave-one-out error estimate because the resulting decision function changed only when SVs were omitted. Thus, a low fraction of SVs would be appropriate for the parameter selection criterion [40].

### 3.4. Feature Extraction and Feature Selection

**Figure 3.**
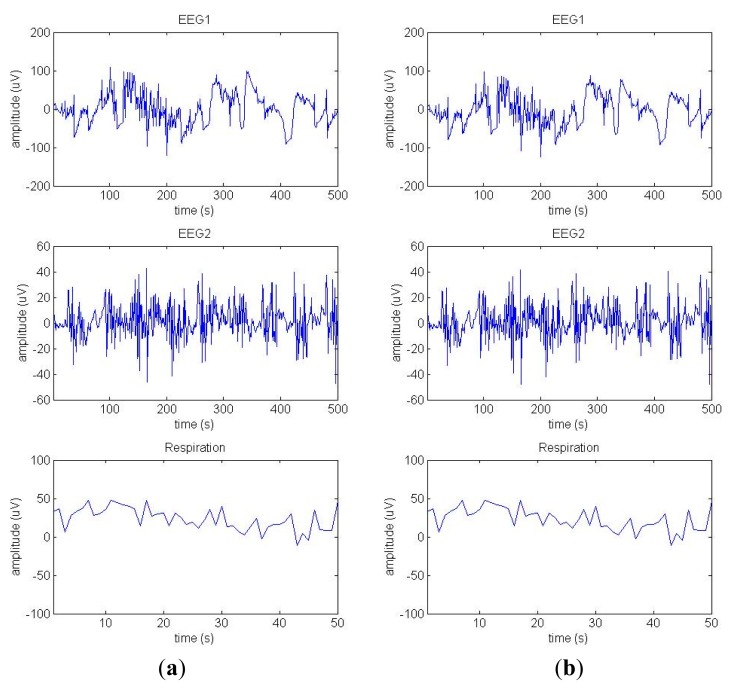
(**a**) Raw EEG1 (Fpz-Cz), EEG2 (Pz-Oz), and respiration signals; (**b**) Filtered EEG1 and EEG2 signals, as well as the respiration signal.

Two differential derivations EEG activity (Fpz-Cz, denoted as EEG1, and Pz-Oz, denoted as EEG2) and a respiration signal are captured in real time, as illustrated in Figure 3a. The raw EEG signals are filtered with a Butterworth bandpass filter with a passband of 0.1–40 Hz [see Figure 3b].

An EEG signal can be characterized by the distribution of the amplitude and time. On the basis of each segment of the EEG signal (128 points per segment), feature parameters can be calculated by statistical analysis. The parameters include the mean, standard deviation, skewness, and kurtosis [19]. On the other hand, the distribution of EEGs and respiration signals in zero and other level crossing intervals can be a very useful alternative feature for drowsiness studies. Parameters in interval analysis are derived from both EEG signals, whereas the regularity of respiration [42] is computed from the respiration signal.

In addition, the EEG1 and EEG2 signals can be converted to the time-frequency domain by adopting a WPT. By referring to the wavelet coefficients listed in Table 2, four distinct frequency bands are derived. The absolute EEG power spectrum for each frequency band is computed by taking the mean absolute value of the corresponding wavelet coefficients. On the basis of Equations (2) and (3), the relative power spectra are calculated as well. By considering the trade-off between the mobile device'scomputational speed and the fatigue detection accuracy rate, Hamming-windowed sub-segments are not adopted in the current study but will be considered for implementation in future research. The performance of the ratios of α, β, θ, and δ activities exhibit a few interesting features to be observed with the changes in the drowsiness level. Thus, four parameters are considered for both referential derivations EEGs [23], which are (α/β), (θ/β), [(θ + α)/β], and [(θ + α)/(α + β)]. Moreover, two additional parameters, namely the center of gravity frequency and frequency variability [43] are obtained on the basis of multiplication of the respective power spectral density frequency values for each frequency band. Table 3 summarizes all of the features extracted and their short descriptions.

As a result, a total of 51 quantitative features (EEG: 25 types of features × two referential derivation EEG activity and respiration: 1 type of feature) were extracted. The EEG and respiration data were labeled with six levels of fatigue denoted as “awake”, “slightly drowsy”, “moderately drowsy”, “extremely drowsy”, “sleep”, and “deep sleep”. However, in this mobile-device-based application, the stages “extremely drowsy,” “sleep,” and “deep sleep” were combined into the same stage because no obvious profiles were found in these stages. Subsequently, the most prominent features were selected with the MI method by using Equations (8) and (10). A comparison was made between the selection solely by MI and the selection by JMI for the 51 features *X* and six-level class *Y*. The output revealed no significant difference between the two types of MI values.

### 3.5. Experiments and Data Acquisition Methodology

Twenty mentally healthy volunteers with no sleeping disorders, no sleep apnea, and no other related illnesses or diseases were recruited for training-data acquisition. Male and female drivers with a mean age of 32 ± 6 years, and from various countries, were included in the study to help understand the variation in features in a better manner. In this study, we only considered healthy volunteers, as drivers with illnesses might contaminate the results. Before the start of the experiment, every volunteer filled out a survey form. Survey questions were related to sleeping hours, time since last awoken, hunger state, mental status, health condition, driving experiences, driving skills, *etc.*, that could directly affect their driving performance. Each volunteer was allowed four hours to become familiar with driving simulation before real experiments were carried out. The actual data gathering experiments were taken continuously for duration of eight hours for two days (daytime, from 11 a.m. to 7 p.m., and night time, from 11 p.m. to 7 a.m.). Thus, for each volunteer, two training samples with driving simulation were acquired, as illustrated in [44]. Video data during the driving session along with real-time EEG activities and respiration signals are recorded to be served as the arousal assessment. The 10–20 international standard of electrode placement was applied [3]. The volunteers' awareness level was marked by expert physicians from our university hospital, as illustrated in previous study [19]. During the alert driving, volunteers are managed to perform driving operation smoothly with many road stimuli, no accidents are to be seen. The volunteers are marked as “slightly drowsy” when volunteers driving speed, as well as the reaction to avoid incoming vehicles in driving simulation are reduced within 5 s interval. The reviews from the physicians' observation indicated volunteers at “extremely drowsy” stage are very unlikely to focus on continuous driving and accidents tend to happened at average of 10 s interval. For the further stages, volunteers are totally lost control with the vehicle operations and some volunteers' vehicles are totally stationary. According to [45], the sleep stages are classified into stage W (wakefulness), stage N1 (NREM 1), stage N2 (NREM 2), stage N3 (NREM 3), and stage R (REM). Among the sleepiness indexes defined in our study, “awake”, “slightly drowsy”, and “moderately drowsy” are three stages derived from stage W in [45], while stage above “extremely drowsy” can be classified as stage N1 in [45].

## 4. Results and Discussion

First, the sixteen most descriptive features obtained by MI are listed in Table 4 in ascending order with the highest MI value on the top of the list. The absolute delta feature in EEG2 is the best primitive feature with the highest MI value, followed by the “TABRATIO” and “TAABRATIO” features. The table also indicates that the frequency-domain analysis of EEG2 comprises the 68.8% majority compared to the EEG1 and respiration signals. Among the four frequency bands, delta activity contributed to 43.8%, indicating its significant fitness as a descriptive feature for fatigue estimation. In addition, the frequency variability also shows promise as a useful fatigue indication feature (31.3%). However, the frequency-band ratio features in EEG2 are apparently better than those in EEG1. The regularity of respiration is another favorable feature to be adopted. In terms of EEG1, it is unlikely to provide a satisfactory feature in comparison to EEG2. The standard deviation of EEG1 determined by statistical analysis reveals the most significant features that can be fed as input features into the SVM.

**Figure 4.**
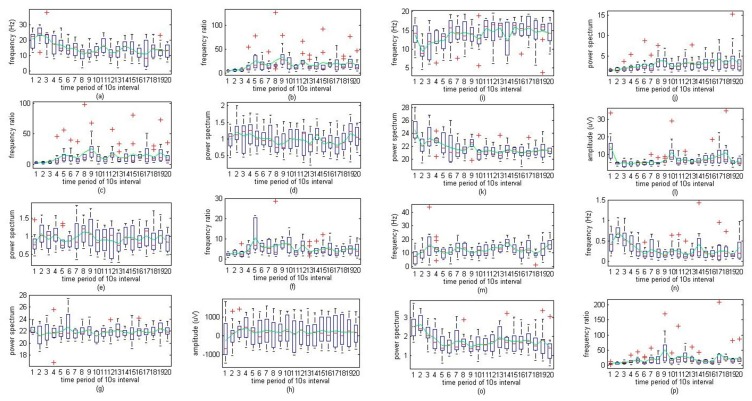
Graphs illustrating a 200-s time segment for volunteer one with 10 s of each of his 16 most descriptive features: (**a**) ABSDELTA EEG2; (**b**) TAABRATIO EEG2; (**c**) TABRATIO EEG2; (**d**) FVBETA EEG2; (**e**) FVBETA EEG1; (**f**) TBRATIO EEG2; (**g**) FVDELTA EEG2; (**h**) RGPNG; (**i**) ABSTHETA EEG2; (**j**) CGFDELTA EEG2; (**k**) FVDELTA EEG1; (**l**) STDEEG1; (**m**) ABSDELTA EEG1; (**n**) RELDELTA EEG2; (**o**) FVALPHA EEG2; and (**p**) TAABRATIO EEG1.

Figure 4a–p displays the 16 most descriptive features for the 13-min period of volunteer 1 (subject 1, male, age 24 years old, Malaysian). The levels indicated are “awake”, “slightly drowsy”, “moderately drowsy”, “extreme drowsy”, “sleep”, and “deep sleep,” denoted as 6, 5, 4, 3, 2, and 1, respectively. The fatigue level was observed and specified by expert physicians during the entire monotonous driving experiments. The *x*-axis indicates consecutive time intervals of 10 s for 200 s (13 min) during monotonous driving. Accordingly, the transition starts at approximately the 3rd time point from awake to drowsy state. The first feature, “ABSDELTA EEG2” tends to decrease when the awareness level begins to slope downwards. In comparison with the previous feature, “TAABRATIO EEG2” reveals a similar increasing trend as the driver became fatigued. “TABRATIO EEG2” also exhibits an increasing slope because of the decreasing trend in beta activity. In contrast, “FVBETA” for both EEG activities exhibits an intermittent increasing pattern as opposed to the “TABRATIO EEG2” feature. Moreover, the ratio between the fast wave and the slow wave (“TABRATIO EEG2”) exhibits a progressively increasing trend as the driver vigilance level declines. Next, the EEG2 delta frequency of variability (“FVDELTA EEG2”) exhibits an increasing trend at each transition phase but slightly increases throughout the same phase. Similarly, the regularity of respiration (“RGPNG”) follows the same pattern. The sharp decreases in “FVDELTA EEG2” and “RGPNG” can be indicated as useful indicators of fatigue levels, similar to “ABSTHETA EEG2”. In the case of “CGFDELTA EEG2,” the activity exhibits an increasing slope as the driver starts to become fatigued. In addition, “FVDELTA EEG1” exhibits a sharp decrease during the increased fatigue state transition. Surprisingly, the standard deviation of EEG1 (STDEEG1) demonstrates an unexpected increase as the driver vigilance level decreases. As for the absolute EEG1 power spectra (“ABSDELTA EEG1”) and relative EEG2 power spectra (“RELDELTA EEG2”) of delta waves, the activities indicate a significant downward sloping trend, and “FVALPHA EEG2” also exhibits a similar decreasing pattern at the beginning when the driver fatigue level is degraded but increases again after the fatigue level increases. The final feature, “TAABRATIO EEG1”, exhibits an opposite trend to that of “FVALPHA EEG2”, in which this activity increases when the driver fatigue level increases and decreases again after the transition state. However, this type of tracking could be a good indicator for predicting the beginning stage of the transition state when the fatigue level is degraded (sleepiness index is increased).

**Figure 5.**
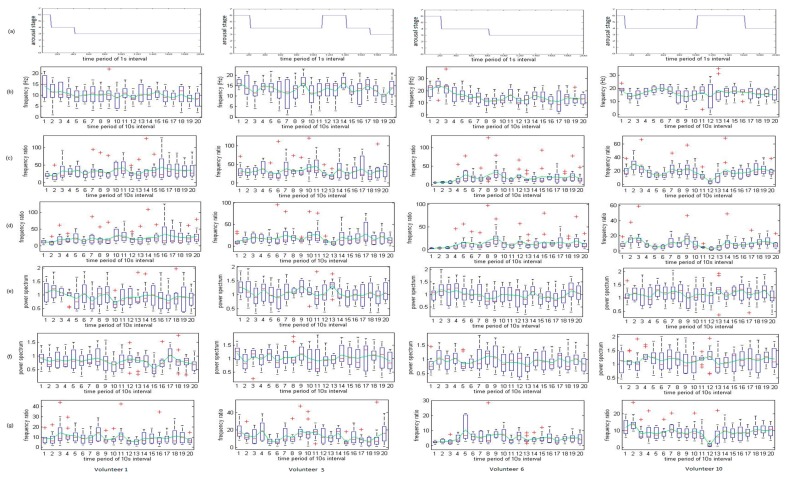
Arousal stage for four different volunteers for the top six most descriptive derived from MI (shown in Table 4) with (**a**) arousal stage with 1 s interval; (**b**) ABSDELTA EEG2; (**c**) TAABRATIO EEG2; (**d**) TABRATIO EEG2; (**e**) FVBETA EEG2; (**f**) FVBETA EEG1; and (**g**) TBRATIO EEG2 with 10 s interval.

The variation in features for volunteers in fatigue detection with different nationalities is further investigated. Figure 5 illustrates the arousal stage for volunteers from volunteer 1 (Malaysia), volunteer 5, (Indonesia), volunteer 6 (China), and volunteer 10 (Korea), respectively for the specific consecutive time intervals of 10 s for 200 s (13 min), as described above. As seen, each volunteer exhibits similar behavior at a specific time period where their arousal stages are in decreasing trend. However, at approximately the 20 s-period, volunteer 5 and volunteer 10 arousal stages are increased even though their initial stage is much lower. This was due to volunteers experienced shock (sudden awake) by the siren from the incoming vehicle in driving simulation. The radical change patterns are further evaluated by examining their respective features variation trends. It is very interesting to observe that most volunteers had a significant reduction in arousal stage going directly into stage 4, skipping the stage 5, after long hours of continuous driving. Indeed, similar patterns are observed in the rest of the volunteers' performance charts. It is very well-noticed that the driver alertness level, after long hours of driving without rest for consecutive periods, can drop dramatically.

Next, the volunteers top six feature variations are observed, as depicted in Figure 5b–g. Based on the arousal stage in Figure 5a for each volunteer, volunteer 1 and volunteer 6'ssimilar patterns of exhibited arousal dropped, while volunteer 5 and volunteer 10 demonstrated similarity in arousal, decreasing for a 200 s time interval. Firstly, based on the previous description of volunteer 1 in how features vary in accordance to the arousal stage, volunteer 5 possessed very high significant patterns, as does volunteer 1, especially in “ABSDELTA EEG2”, “TAABRATIO EEG2”, “TABRATIO EEG2”, “FVBETA EEG2”, and “TBRATIO EEG2”. The fifth feature “FVBETA EEG1” demonstrated an identical trend in the first half portion of feature variation, but was slightly different near the end portion. The volunteer 1 “FVBETA EEG1” showed rapid increased at the 17th time period while volunteer 6 showed low variations when both volunteers are in stage 3 (extremely drowsy state). In fact, very low variation in “FVBETA EEG1” in volunteer 6 depicted an ideal result, demonstrating that arousal stage dropped. On the other hand, “ABSDELTA EEG2” of volunteer 5 and volunteer 10 presented a high correlation with the increase of arousal, and “ABSDELTA EEG2” tends to increase as well. It matches the profiles, similar in volunteers 1 and 6, discussed above. The same characteristic is also accurately demonstrated in features “TAABRATIO EEG2”, “TABRATIO EEG2”, “FVBETA EEG1”, and “TBRATIO EEG2”. In the case of the fourth feature, “FVBETA EEG2”, there is a rapid increasing trend in 13th period for volunteer 5, but not observed in volunteer 10, which is believed to be the ideal match to the arousal stage change. Convincingly, even though the volunteers had different nationalities; their EEG activity is highly correlated and illustrates the relationship between arousal stages and their respective features.

Overall, it is observed that the features extracted from EEG2 exhibit better results in comparison with the EEG1 extracted features. In the case of the frequency-band-ratio computation, the mutual integration of alpha activity and theta activity produces a more promising effect than alpha activity or theta activity alone. From the graphs, it is observed that beta activity, indeed, exhibits a significant drop when the driver fatigue level increases. Additionally, beta activity exhibits a sudden drop when the driver' vigilance level is considered to be decreased during monotonous driving. Thus, all ratios involving beta activity exhibit increasing sloping trends as the driver becomes fatigued. For both EEGs, the delta wave in the absolute power spectra, the relative power spectra, the center of gravity frequency, or frequency variation, demonstrate significant positive correlations with the driver awareness level (the feature values decrease). Therefore, the delta wave alone can be a useful indicator for driver fatigue measurement.

These selected features served as input parameters for the SVM classifier. The use of the SVM classifier is intended to classify the current state of the driver into six-level categories in a real-time situation. To ensure the accuracy of the SVM estimation rate, the current study also investigated the influence of the optimum feature size used for training and testing. The total training data (20 volunteers × 2 samples each × 8 h each = 320 h of samples) collected were divided into training sets and testing sets. The training sets consisted of 70% from individual samples (a total of 224 h), whereas the testing sets were generated from the remaining 30% of individual samples (a total of 96 h).

**Figure 6.**
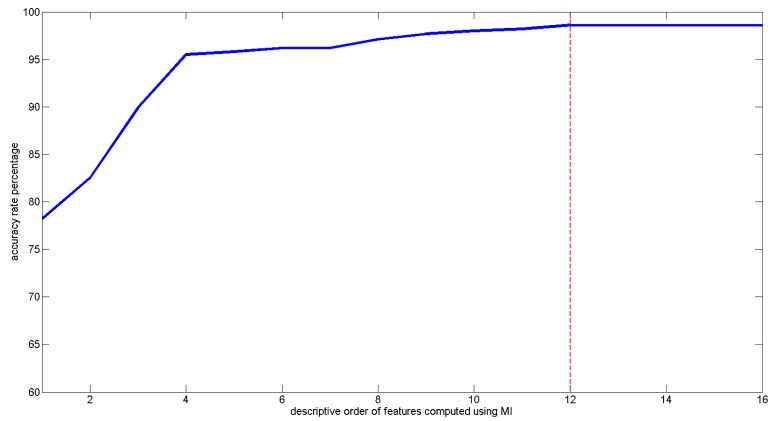
Accuracy rate of the SVM classifier corresponding to the number of features used to predict the driver awareness level. The choice and number of features adopted are based on the rank of the most descriptive features in Table 4 in descending order.

The resulting graph is depicted in Figure 6. As stated, the accuracy of the SVM increases at an average rate of 2.5% with the addition of features. The increase in accuracy halts when more than twelve features are employed to train and test the SVM classifier. Therefore, we concluded that twelve features would be the optimum number of the most descriptive features to be used in the SVM classifier when the accuracy rate reached up to 98.6%. By further improving the reliability of our proposed mobile application, the sensitivity and specificity [18] were derived as well. The SVM classification performance for estimating six-level sleepiness onset has 98.6% accuracy, 99.1% sensitivity, and 99.5% specificity.

**Figure 7.**
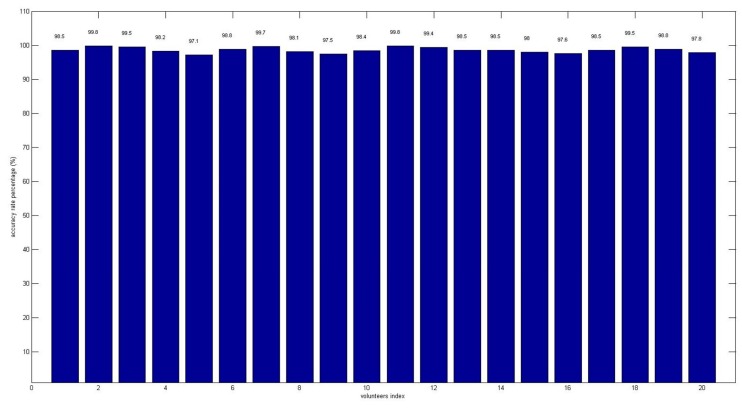
Accuracy rate of the SVM classifier corresponding to the twelve features (optimum) used to predict the driver awareness level for each volunteer.

However, there is a constraint needed to be taken into consideration, which is the computational complexity of SVM classifier to indicate the driver arousal state in mobile-device. Indeed, the number of support vectors obtained from the SVM trained model is a critical condition. The least support vectors required, the lower computation required by SVM classifier. In fact, the total numbers of support vector obtained are 2,689,021 from trained model, which is not an optimal solution to be deployed in mobile-device. Thus, extracting 30% of most relevant support vectors (806,706) to be developed in mobile-device provided exactly the same accuracy rate in driver arousal stage prediction. The accuracy rate results for arousal stages prediction in mobile-device by applying SVM classifier in each volunteer are shown in Figure 7. As depicted in the figure, accuracy rate by volunteer 5 is the lowest, at approximately 97.1%, while volunteer 2 had the highest accuracy rate at 99.8%. The accuracy rate to predict the arousal level for twenty volunteers averages at 98.5% ± 1.4%. In fact, no extra independent processing unit and display modules are required, as both modules take place in the mobile device. Our proposed method allowed the system to transfer from one vehicle to another, portably, without causing any inconvenience to the user.

**Figure 8.**
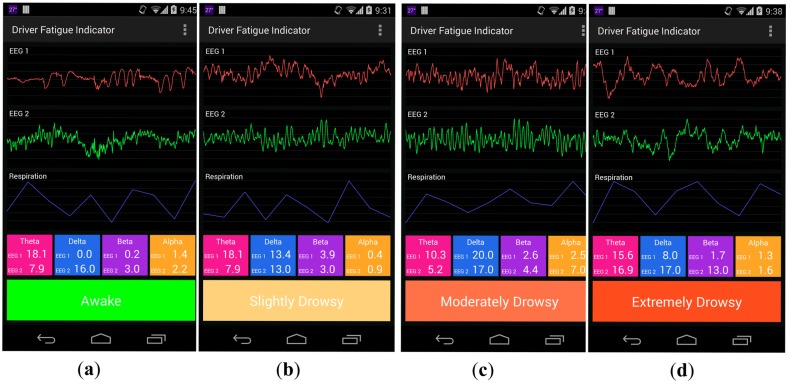
Sample screenshots of the four distinct driver sleepiness levels: (**a**) wakeful state (level 6); (**b**) slightly drowsy state (level 5); (**c**) moderately drowsy state (level 4); and (**d**) extremely drowsy (level 3).

An SVM model with parameters was obtained once the RBF-based kernel SVM training was completed. The mobile device utilized such a model to estimate the driver fatigue level. Figure 8 shows four different sleepiness levels encountered on the basis of the twelve most descriptive extracted features from the EEG1, EEG2, and respiration signals. The uppermost and middle parts of the graph correspond to the raw EEG signals (at a sampling rate of 100 Hz) obtained from the two referential derivations Fpz-Cz and Pz-Oz regions, respectively, whereas the bottom part of the graph corresponds to the respiration signal at a sampling rate of 1 Hz. The four frequency-band (α, β, θ, and δ) values for the EEGs are derived by the WPT method and presented in the color columns below the graphs.

Table 5 illustrated different classifiers utilized or proposed by other researchers in estimating driver alertness level. The KPCA-SVM had the highest accuracy rate among all the classifiers. The foremost reason is the adoption of PCA algorithm for features dimension reduction before serve as inputs to the SVM classifier. However, its increment in accurate prediction rate is only 0.3% higher than the SVM classifier in this study, which is not a huge influential. Furthermore, addition of features dimension reduction increased the computation complexity and more time-consuming to be employed in mobile device. Other classifier, such as Fuzzy, although performed faster, its accuracy of prediction rate is a disappointment which is 85.52%. The same characteristic is also applied to Bayesian technique. In contrast, artificial neural network perform worse than the SVM algorithm, even though it possess the capability of self-learning as more datasets are fed in. Therefore, in this study, SVM classifier with most relevant support vectors extracted from SVM trained model can perform well with high accuracy rate to predict driver alertness level in real-time. Future work may include exploring more appropriate relevant algorithms including frequency extraction method and feasible classifier to predict driver drowsiness using wearable devices.

## 5. Conclusions

A real-time long-term mobile-based driver-fatigue-monitoring system is proposed and developed. The multichannel sensor module consists of eight-channel EEG (two referential derivations) and respiration sensors that transmit their raw data to a mobile device via Bluetooth wireless communication. The mobile application filters the received raw EEG and respiration signals and subsequently decomposes the filtered signals into four discrete frequency bands: α, β, θ, and δ. The most descriptive features are extracted and serve as input features for the SVM classifier. The SVM classifier is trained using a desktop computer, and a successive model is adopted in the mobile device to estimate driver sleep-onset events in real time. Conclusively, the study indicates that EEG signals in the Pz-Oz region serve as a higher impact criterion for fatigue detection than EEG signals in the Fpz-Cz region, providing approximately 69% of the total features. Among the four frequency bands, a sole delta wave is a superb fatigue indicator, whereas the mutual addition of alpha, beta (fast), and theta (slow) waves exhibit a greater satisfactory effect compared to alpha, beta, and theta waves alone. In addition, a frequency variation analysis shows that it is worthy as one of the promising features in the top sixteen descriptive features. Overall, an accuracy rate of up to 98.6% can be achieved using a test with the optimized top twelve features selected by the MI algorithm. The authors would like to investigate the interaction dynamics between oscillations generated by different neuronal population with wavelet coherence suggested by [45].

## Figures and Tables

**Table 1.** Specifications of the sensor module that consists of an EEG sensor, a respiratory sensor, a microprocessor, and a Bluetooth module.

**Module**	**Components**	**Specifications**
EEG sensor [24]	sensors	14 saline sensors
	sampling rate	100 Hz
respiration sensor [25]	sampling rate	1 Hz
microprocessor [26]	ADC	10 bit
	size	7.75 × 0.65
Bluetooth module [27]	maximum transmission range	106 m
	average power consumption	75 mW, at 3.3 V
power	battery	4.5 V
Google Nexus 5 [28]	screen	4.95″ 1920 × 1080 display
	dimension	69.17 × 137.84 × 8.59 mm
	operating system	Android 4.4.2
	wireless	Dual-band Wi-Fi (2.4 G/5 G)
		Bluetooth 4.0

**Table 2.** Frequency bands extracted from the wavelet coefficients and grouped into corresponding frequency bands. The respective frequency bands are derived by finding the mean of the respective wavelet coefficient vector.

**Wavelet Coefficients**	**Frequency Range**	**Frequency Bands**
W_6,1_–W_6,5_	0 Hz–4 Hz	Delta
W_6,6_–W_6,10_	4 Hz–8 Hz	Theta
W_6,11_–W_6,17_	8 Hz–13 Hz	Alpha
W_6,18_–W_6,45_	13 Hz–20 Hz	Beta

**Table 3.** Extracted features from filtered EEG signals and respiration signals that can be categorized by (**a**) statistical analysis; (**b**) interval analysis; and (**c**) frequency analysis.

**Analysis**	**Features**	**Description**
statistical	MEEG	Mean value: $\bar{x}=\frac{1}{n}\sum_{i=1}^{n}x_{i}$
	SDEG	Standard Deviation: $\sigma_{x}=\sqrt{\frac{1}{n}\sum_{i=1}^{n}{\left(x_{i}-\bar{x}\right)}^{2}}$
	SKEEG	Skewness: $s=\frac{\frac{1}{n}\sum_{i=1}^{n}{\left(x_{i}-\bar{x}\right)}^{3}}{\sigma_{x}^{3}}$
	KREEG	Kurtosis: $k=\frac{\frac{1}{n}\sum_{i=1}^{n}{\left(x_{i}-\bar{x}\right)}^{4}}{\sigma_{x}^{4}}$
interval	ZCEEG	Zero-Crossing: number of zero-crossings in a signal
	RGPNG	Regularity of Respiration: computing the second peak in the autocorrelation function from the respiration signal [41]
frequency	ABSDELTA	Absolute PSD for Delta Band
	ABSTHETA	Absolute PSD for Theta Band
	ABSBETA	Absolute PSD for Beta Band
	ABSALPHA	Absolute PSD for Alpha Band
	RELDELTA	Relative PSD for Delta Band
	RELTHETA	Relative PSD for Theta Band
	RELBETA	Relative PSD for Beta Band
	RELALPHA	Relative PSD for Alpha Band
	ABRATIO	ABSALPHA/ABSBETA
	TBRATIO	ABSTHETA/ABSBETA
	TABRATIO	(ABSTHETA + ABSALPHA)/ABSBETA
	TAABRATIO	(ABSTHETA + ABSALPHA)/(ABSALPHA + ABSBETA)
	CGFDELTA	$\text{CGF}=\frac{\sum_{i}P(f_{i})\times f_{i}}{\sum_{i}P(f_{i})}$
	CGFTHETA	
	CGFALPHA	
	CGFBETA	
	FVDELTA	$FV=\frac{\sum_{i}P(f_{i})\times f_{i}^{2}-\frac{{\left(\sum_{i}P(f_{i})\times f_{i}\right)}^{2}}{\sum_{i}P(f_{i})}}{\sum_{i}P(f_{i})}$
	FVTHETA	
	FVALPHA	
	FVBETA	

**Table 4.** Sixteen most descriptive features obtained by MI.

**No.**	**Features**	**No.**	**Features**
1	ABSDELTA EEG2	9	ABSTHETA EEG2
2	TAABRATIO EEG2	10	CGFDELTA EEG2
3	TABRATIO EEG2	11	FVDELTA EEG1
4	FVBETA EEG2	12	STDEEG1
5	FVBETA EEG1	13	ABSDELTA EEG1
6	TBRATIO EEG2	14	RELDELTA EEG2
7	FVDELTA EEG2	15	FVALPHA EEG2
8	RGPNG	16	TAABRATIO EEG1

**Table 5.** Comparison of classifiers in predicting the driver's vigilance level.

**Classifier**	**Dataset**	**Accuracy (%)**
KPCA-SVM [2]	Thirty-channels EEG, one-channel ECG and vertical EOG from	98.8
Fuzzy [4]	PERCLOS, eye closure duration, blink frequency, nodding frequency, face position, fixed gaze	85.52 (average)
Fuzzy Neural Network [9]	Thirty-three-channels EEG	88 (correlation)
Artificial Neural Network [10]	Midline sites, one central (Cz), and other midway between parietal and occipital sites (Pz/Oz)	94.4
Bayesian [12]	EEG-MPF, EEG-α/β, RRV3, tracking error	87.5
SVM-RFE [21]	EEG, EOG	75
SVM(desktop PC)	Two referential derivation EEG activity (8-channels) and respiration signals with total number of support vectors from train model	98.6
SVM-Mobile(Proposed)	Two referential derivation EEG activity (8-channels) and respiration signals with extracted 30% total number of support vectors from the trained model	98.5
